# Transient cyanobacteria drive hydrostatic photogranulation but disappear during microbial succession

**DOI:** 10.1016/j.bioflm.2025.100339

**Published:** 2025-12-01

**Authors:** Sandra Galea-Outón, Jérôme Hamelin, Kim Milferstedt

**Affiliations:** INRAE, Univ Montpellier, LBE, 102 Avenue des étangs, Narbonne, 11100, France

**Keywords:** Community succession, Community assembly, Microbial engineering, Keystone species, Bioaugmentation

## Abstract

Photogranulation transforms activated sludge into spatially organized photogranules, yet the role and fate of cyanobacterial strains that trigger this process remain poorly understood. Here, we tested the ability of seven strains of filamentous cyanobacteria to induce photogranule or mat morphotypes in replicated hydrostatic incubations. Despite successful granulation in all replicates when granule-forming strains were added, sequencing revealed that these keystone cyanobacteria responsible for photogranulation were outcompeted during community succession and absent from mature photogranules. A time-series experiment demonstrated that the added granule-forming strain *Geitlerinema* sp. A4 dominated the cyanobacterial community early on but was gradually replaced by mat-forming taxa, such as *Tychonema* and *Planktothrix*. Nevertheless, the granule morphotype persisted even after the founder strain vanished. These findings highlight that transiently dominant organisms can act as microbial engineers, steering ecosystem trajectories without remaining in the final community. This study refines our understanding of microbial succession and has practical implications for selecting the inoculum in biotechnological processes using photogranules.

## Highlights

• Replicated photogranulation from activated sludge was achieved by bioaugmentation.
• Photogranules form in a week, but cyanobacterial succession continues for 3 weeks.
• Photogranulating cyanobacteria are outcompeted shortly after incubation.
• Cyanobacteria from the order *Geitlerinematales* may promote photogranulation.

## 1 Introduction

The diverse microbial community of activated sludge contains a wide range of microorganisms that thrive under extremely contrasted environmental conditions [1]. These organisms may enter the wastewater treatment plant with the used water and carry the signature of human activities or of the collection system, or they may be indigenous in the various unit operations of wastewater treatment. Even the air used in mechanical aeration of activated sludge basins may be a source of a specific microbial contribution to the community, like phototrophic microorganisms [2]. These organisms may be present in only small quantities in the sludge and may not actively grow in a wastewater treatment plant. However, changes in environmental conditions may strongly induce their activity and set off the development of the ecosystem on a distinct trajectory. If activated sludge is left unagitated for several days in a confined environment, an anaerobic community develops, dominated by organisms from the anaerobic food web. If light is added to the aforementioned conditions, the activated sludge may transform into sphere-like phototrophic aggregates in a process called hydrostatic photogranulation [3]. Hydrostatic photogranulation is influenced by the physico-chemical environment, e.g., the nutrient composition and the availability of divalent cations like Ca^2+^ [4]. However, also the biotic composition of the activated sludge may play a role. We test here how the addition of small amounts of microorganisms, notably filamentous and motile cyanobacteria, can profoundly change the developmental trajectory of an ecosystem towards a spatially explicit ecosystem. Filamentous and motile cyanobacteria are believed to be the ecosystem engineers inherent to, or added to the activated sludge community that push the system towards an unexpected climax state of ecosystem development. The ability of filamentous cyanobacteria to engineer their surroundings by creating spatial structures caused by EPS secretion and resulting gliding motility has been shown experimentally and in mathematical models [5,6].

Photogranules could be obtained from diverse environments [[7], [8], [9], [10]]. The phenomenon is therefore rather common, if appropriate conditions are applied. During hydrostatic photogranulation, the biomass undergoes several discernible states characterized by biomass compaction, contraction and consolidation until a mature photogranule of several millimeters in diameter is formed. The final size of the photogranules depends on the sludge volume [11]. These changes in morphology are visibly accompanied by changes in the community composition, most notably by a massive growth of phototrophic organisms. The enrichment of phototrophic organisms during static incubation can be quantified by an increase of chlorophyll *a* concentration [12] or seen in the abundance of phototrophic sequences in rRNA amplicons that may reach on average 40 % by the end of the process [13].

The mechanisms behind the emergence of this physical structure is not known yet, but several factors may play a role. Most of these factors are related to the nature of filamentous cyanobacteria, like the strength of extracellular polymeric substances [14], their gliding motility [15] or their reaction to predation by microbial grazers [16]. While filamentous cyanobacteria are suspected to be essential for the formation of photogranules [8], not all filamentous cyanobacteria appear to promote photogranulation [13]. Since filamentous cyanobacteria in activated sludge are rare (<1 % of all bacterial cells) [13], it may be that an incubated aliquot of activated sludge does not contain a photogranule-forming cyanobacterium in sufficient quantities, or that non-photogranule forming cyanobacteria become dominant. As a consequence, under controlled laboratory conditions, consistent photogranulation is not guaranteed for every biological replicate of activated sludge. The morphotypes found after incubation range from spherical to mat-like and hemispheroids aggregates [9,13], similar to what is observed when sludge is exposed to different physico-chemical conditions [4].

Augmenting the initial activated sludge with monoclonal isolates of cyanobacteria isolated from mature photogranules changed the success rate of forming a certain morphotype: higher proportions of photogranules or microbial mats were observed in repeated incubations. The augmented cyanobacteria belong to Subsection III in Bergey's Manual of Systematics of Archaea and Bacteria which are non-heterocystous, with unbranched filaments, most of which exhibit gliding motility [15]. Surprisingly, the added cyanobacteria believed to lead to the development of a photogranule morphotype became rare in mature photogranules. It was hypothesized that it was replaced in a microbial succession along the trajectory towards a photogranules morphotype but that it left a lasting effect on the spatial development of the microbial aggregate [13]. To test this hypothesis, sacrificial samples need to be taken at different time points in replicated incubations, if the final morphotype that would develop in each vial could be predicted with certainly. However, because of the presence of a distribution of various morphotypes at the end of the incubation, sampling a time-series had not been attempted, as the interpretation of intermediate time points was impossible [13].

Here, we optimized the experimental approach by Joosten et al. [13] so that we can now predict with confidence the morphotype of replicated incubations for hydrostatic photogranulation. This allows us to sample biomass at intermediate states of morphotype development along its trajectory towards a predetermined morphotype. The analysis of a time series of microbial communities, notably the cyanobacterial population, can then be interpreted in the light of microbial succession. We show the succession of the added cyanobacteria, responsible for photogranulation: when it disappears from the microbial community during hydrostatic photogranulation and its relation with morphology succession.

## 2 Materials and methods

### 2.1 Set-up of hydrostatic photogranulation

The capacity of an activated sludge matrix to form a photogranule was tested as a function of augmenting it with different monoclonal cyanobacterial strains. Photogranulation was quantified by time-lapse imaging and automated image analysis, notably by the contraction of the biomass, as observed through the bottom of the vials in which the mixture was incubated. Biomass contraction is a proxy for photogranulation. For this, vials were placed on a desktop scanner and images of all vial bottoms were acquired in 8-h intervals. The detailed protocol for the incubations and image analysis is published in Joosten et al. [11]. 72 vials with a volume of 4 mL were followed in parallel. Replication was essential to the experiments because of the risk of developing a distribution of morphotypes rather than a unique morphotype per incubation condition. The non-invasive monitoring of high numbers of replicates in parallel with relatively large individual areas of 113 mm^2^ is possible with a scanner. However, it comes at the cost of resolution and sharpness of the resulting images, compared to low-throughput but more highly resolved methods using, e.g., optical coherence tomography or confocal imaging. This compromise is acceptable for this study. Each vial was filled with 2 mL of activated sludge or a mixture of homogenized cyanobacterial culture and activated sludge. The vials were exposed to a constant light intensity of 70 μmol m^−2^ s^−1^ photosynthetically active radiation (PAR) measured at the top of the vials. The light was emitted from a cold white LED plate placed above the set-up. The vials were placed into laser-cut holes of a black-anodized aluminum grid with a thickness of 1.5 mm. The outer diameter of the vials matched the diameter of the holes. The grid itself was placed on the document table of the scanner. The holes allowed the image acquisition of the bottoms of the vials by scanning. The remainder of the document table was obscured by the grid to avoid stray light.

### 2.2 Biological material

Fresh conventional activated sludge was collected from the aeration basin of two urban wastewater treatment plants, ‘Sludge 1’ at the village of Ornaisons, France (2000 person equivalents) and ‘Sludge 2’ at the city of Narbonne, France (120000 person equivalents). Samples were taken at different periods of the year over two years. The mean concentration of activated sludge was 3.4 ± 1.8 g TSS·L^−1^. Two sludge sources were used to assess a putative effect of sludge source on photogranulation.

To the activated sludge, we added small amounts of xenic strains of filamentous motile cyanobacteria from our strain collection. The strains *Planktothrix* sp. (strain F9), *Nodosilinea* sp. (strain F12), *Tychonema* sp. (strain F1), *Tildeniella* sp. (strain F16), *Kamptonema* sp. (strain F10), and *Geitlerinema* sp. (strains A4 and A5) were previously isolated from mature photogranules. The cyanobacteria were cultured in 50 mL flasks with 30 mL of BG-11 medium under a day/night light regime of 16/8 h at 10 μmol m^−2^ s^−1^ PAR.

For the reproducibility of the augmentation experiments in the relatively small incubation volumes of 2 ml, it was essential to ensure comparable concentrations of cyanobacteria in each vial. Achieving this is technically challenging, because it is difficult to reproducibly detach biomass portions of comparable size from mat- or photogranule-morphotypes of monoclonal cyanobacterial cultures. The dense and interwoven filamentous matrix prevents separating equal-sized pieces using standard pipetting. To overcome this limitation, we used an industrial disperser (T-25 Ultra-Turrax, IKA Werke GmbH, Staufen, Germany) to prepare a homogenized slurry from the morphologically developed cyanobacterial cultures. Cultures were homogenized for 1 min at 11000 rpm. Longer times or higher rotational speeds reduced cell viability, whereas shorter times did not achieve sufficient homogenization for reproducible pipetting. The resulting slurry was mixed with activated sludge before aliquoting into individual vials.

### 2.3 Experimental plan

In a typical experiment, 54 vials were incubated with sludge augmented with a slurry of one cyanobacterial strain. 18 vials served as control and contained only activated sludge (Experiments 1 to 9, Table 1). In Experiments 1 to 9, 100 % of the biological replicates developed the same final morphotype. As a consequence, a reduced number of vials was used in the following experiments. In Experiments 10 and 11, all seven cyanobacterial strains were added individually to 9 vials per strain. The average duration of an experiment was 23 ± 5 days.

**Table 1:** Summary of the conducted experiments. The availability of sequenced 23S rRNA amplicons at the beginning (b), at intermediate time points (i) and at the end (e) is indicated in the last column.

Experi-ment	Duration [d]	Sludge + Strain(s)		Morpho-type	Numbers of vials		23S amplicons available
					controls	augmen-tations	
1	21	1	A4	granule	18	54	
2	23	1	A4	granule	18	54	b/i/e
3	12	1	A5	granule	18	54	
4	26	1	F10	granule	18	54	
5	21	2	F10	granule	18	54	
6	25	1	F1	mat	18	54	
7	21	1	F9	mat	18	54	
8	28	1	F12	mat	18	54	
9	30	1	F16	mat	18	54	
10	21	1	all strains	both	9	9 per strain	b/e
11	24	2	all strains	both	9	9 per strain	

For experiment 10, one sample was taken of the untreated activated sludge and the augmented activated sludge prior to incubation. At the end of every experiment, when no more changes in morphotypes were observed over several days, two samples were taken from the now incubated untreated activated sludge (‘Control’) and from the incubated augmented sludge.

In Experiment 2, in which Sludge 1 was augmented with the strain *Geitlerinema* sp. A4*,* the putative succession of cyanobacteria during photogranulation was followed at intermediate time points. In addition to the typical samples at day 0 and at the end of the experiment (here 23 days), additional samples were taken on days 1, 3, 7, 10 and 16. Three to six samples of augmented vials and one or two samples of controls were taken at each time point for sequencing.

### 2.4 Sequence generation

The biomass formed in the vials could be either a photogranule or in the most extreme case remained an unconsolidated biomass resembling activated sludge even after incubation. Entire photogranules and mats were sampled for community analysis when available, otherwise, 500 μL of vial content were sampled when the biomass was unconsolidated. Genomic DNA was extracted using the FastDNA Spin Kit for Soil (MP Biomedicals, USA) according to the manufacturer's recommendations and stored at −20 °C before use.

23S rRNA gene amplicons were generated using the primers pair p23SrV_f1 (5′-GGACAGAAAGACCCTATGAA-3′) and p23SrV_r1 (5′-TCAGCCTGTTATCCCTAGAG-3′) [17] targeting phototrophs. The PCR mixtures as above were used. A Mastercycler X50s thermal cycler (Eppendorf, Germany) was used as follows: 98 °C for 2 min, followed by 30 cycles of 98 °C for 20 s, 59 °C for 30 s, and 72 °C for 30 s, with a final extension at 72 °C for 10 min. The respective lengths of the PCR products were quality-checked using a Bioanalyzer 2100 (Agilent, USA).

In a second PCR of 12 cycles, an index sequence was added. The resulting PCR products were purified and loaded onto the Illumina MiSeq cartridge according to the manufacturer's instructions for sequencing of paired 300 bp reads (v3 chemistry). Library preparation and sequencing was done at the GeT PlaGe Sequencing Center of Toulouse, France (https://get.genotoul.fr/). Sequences used in the manuscript are available at the European Nucleotide Archive under the accession number PRJEB91337. Samples that were used to produce Fig. 3, Fig. 4 are listed in Table S 1. Sequence cleaning and quality control were done as in Joosten et al. [13] using the modified standard operational procedure for Mothur 1.48.0 [18]. We used Operational Taxonomic Units (OTUs) grouped at 0 % dissimilarity for the analysis.

Amplicons were sequenced for samples from experiments 2 and 10 (Table 1). The data presented here are based on 23S rRNA amplicons from the initial activated sludge samples and at the endpoints of the incubations. In experiment 2, additional samples at days 1, 3, 7, 10, 16 were sequenced. Altogether, the analysis of 23S rRNA sequences yielded up to 39 cyanobacterial OTUs. For plotting, we disregarded minor cyanobacterial OTUs and only visualized major OTUs. These are identified at the genus level when possible and expressed as relative abundance of the cyanobacteria population. Note that a change in relative abundance may be related to a change in absolute abundance of the OTU, to a change in absolute population size, or a mix of the two.

### 2.5 Statistical analyses

The data were analyzed using R 4.2.3 (R Core Team, 2023). Sequencing data were treated with the R package phyloseq v8.4 [19].

## 3 Results and discussion

In the following, we first describe, how to achieve uniform and reproducible results for an incubation set, i.e., uniform morphotypes at the end of the incubation and comparable trajectories of morphogenesis with time. The two elements are indispensable for the characterization of a succession using sacrificial samples. Then we compare endpoint communities and describe the microbial succession during photogranulation.

### 3.1 Reproducible final morphotypes after addition of granule- and mat-formers

We tested seven xenic, monoclonal strains of cyanobacteria for their ability to transform an allochthonous matrix like activated sludge into a photogranule. To do so, activated sludge was augmented with cyanobacterial isolates in 11 independent experiments (Table 1). The activated sludge was sampled from two different urban wastewater treatment plants. The activated sludge itself did not develop photogranules in any of the altogether 162 incubated control vials, but growth of phototrophic organisms was observed also in these controls. We did not consider seasonality in our sludge samples. We confirm here that not all sludge sources possess the necessary properties to be transformed into hydrostatic photogranules in unaugmented incubations [3]. Differences in the physico-chemical composition of the activated sludge may be part of the reason why certain sludge types do not readily form photogranules [4]. As we show here, the biological composition of the activated sludge may also play a crucial role.

**Fig. 1:**
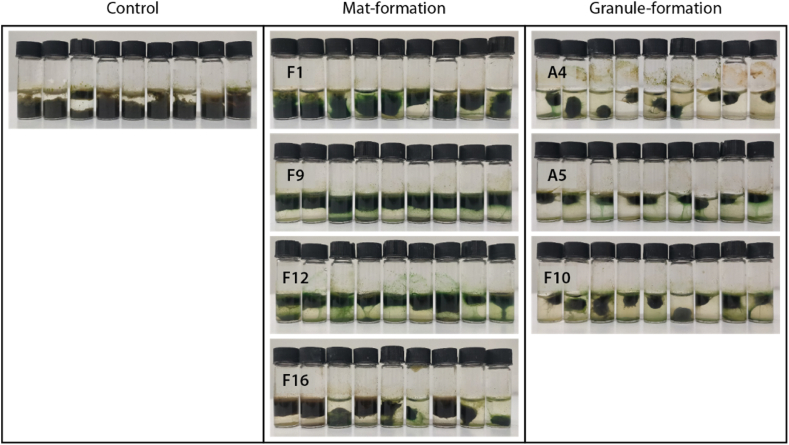
Final morphotypes after 24 days of incubation (Experiment 11 in Table 1). Control vials with unaugmented activated sludge only remained unconsolidated (A), whereas the augmentation with F1, F9, F12 and F16 led to the formation of a mat morphotype (B). The augmentation with A4, A5 and F10 yielded hydrostatic photogranules (C). Note that all replicates gave comparable results.

Phototrophic microorganisms are not part of the activated sludge core microbiome [1], but are part of the sub-dominant microflora of activated sludge and may typically have been airborne during aeration phases of wastewater treatment [2]. In this study, after the cyanobacteria were added to the initial sludge, a single morphotype consistently developed in all replicated vials after augmentation, corresponding to either photogranules or microbial mats, depending on the added strain. The reproducibility of the final morphotype appears to depend on the concentration of added cyanobacteria. Lower concentrations were added in Joosten et al. [13], as cyanobacterial slurries were diluted, leading to less predictable outcomes. We did not quantify the absolute increase of cyanobacterial biomass compared to Joosten et al. [13], but roughly, an at least five-fold higher biomass was added. Fig. 1 illustrates the visual reproducibility of morphotype development, using Experiment 11 as an example. All nine replicates per treatment yielded comparable morphotypes by the end of incubation. In the untreated control (Fig. 1), the resulting phototrophic biomass remained loose and was easily resuspended with gentle agitation, indicating a lack of cohesion, despite the presence of phototrophs. The morphotype that developed after augmentation with mat-forming cyanobacteria appeared visually similar to the control series (Fig. 1) but was consolidated and resistant to resuspension. We refer to this morphotype as microbial mat. Unlike photogranules, microbial mats consolidate over time but do not contract. By the end of the incubation, the biomass still covered nearly the entire horizontal surface of the vial.

Joosten et al. [13] demonstrated that the ability to form a certain morphotype can be predicted from the strains’ growth behavior in the synthetic standard medium BG11, commonly used for growth of cyanobacteria. They used four of the seven strains that were tested here and classified them as photogranule-former (*Kamptonema* sp. F10), hemispheroid-former (*Planktothrix* sp. F9) and two mat-formers (*Nodosilinea* sp. F12 and *Tildeniella* sp. F16). To simplify the classification, we do not differentiate here between the somewhat ambiguous hemispheroid and mat morphotypes. The behavior of the previously tested strains was confirmed in this study (Fig. 1). Three additional strains were used for the first time in this study. Two of them produced hydrostatic photogranules (the strains *Geitlerinema* sp. A4 and A5) and one produced a mat-like morphotype (the strain *Tychonema* sp. F1) as seen in Fig. 1. Also, this behavior is in line with the morphotype development of the strains when grown in the synthetic medium BG11.

The photogranulation property does not correlate with the microscopic phenotype of the cyanobacteria. Contrary to previous studies [20], flexional and gliding motility of the strains were independent of aggregation properties. In addition to their different taxonomy, the granule-former strains differ significantly in their pigmentation and filament width, i.e., F10 appears brownish with a filament width of 5.6 ± 0.4 μm while A4 and A5 are dark green with a filament width of 1.9 ± 0.2 μm and 1.7 ± 0.2 μm respectively (Figure S 1). The morphologies of the seven isolated strains when grown in the synthetic medium BG11 are also shown in the supplemental figure.

### 3.2 Reproducible temporal dynamics of photogranulation

**Fig. 2:**
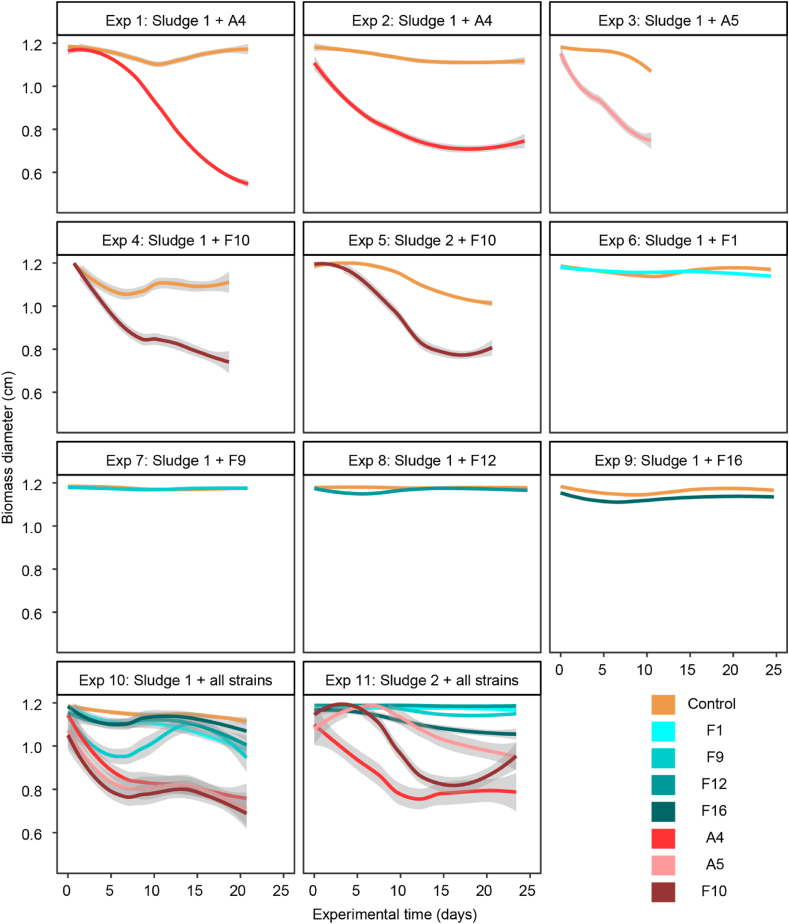
Monitoring biomass contraction of activated sludge during its transformation into a phototrophic community by time-lapse imaging. When granule-former strains were added, contraction was observed, leading to a decrease in equivalent diameter from 1.2 cm to below 0.9 cm. Uncontracted samples remain larger than 1 cm. The shaded areas represent 99 % confidence intervals. In each experiment, 72 vials were incubated: Experiments 1 to 9 comprise 54 replicated augmentations and 18 unaugmented controls. In experiments 10 and 11, the control and each of the seven augmentations was replicated nine times.

Contraction is indicative of hydrostatic photogranulation and can be quantified by tracking biomass coverage of the bottom of the vials over time using time-lapse imaging and automated image analysis. Using this approach, the temporal dynamics of morphotype development in each experiment were followed and plotted in Fig. 2. An equivalent diameter matching that of the bottom of the empty vial (1.2 cm) indicates no contraction, as observed for unconsolidated biomass and microbial mats in Fig. 2, notably in augmentations with F1, F9, F12 and F16. In contrast, during photogranulation, the average equivalent decreases and reaches on average 0.7 ± 0.1 cm by the end of the experiment, as seen in augmentations with A4, A5 and F10.

**Fig. 3:**
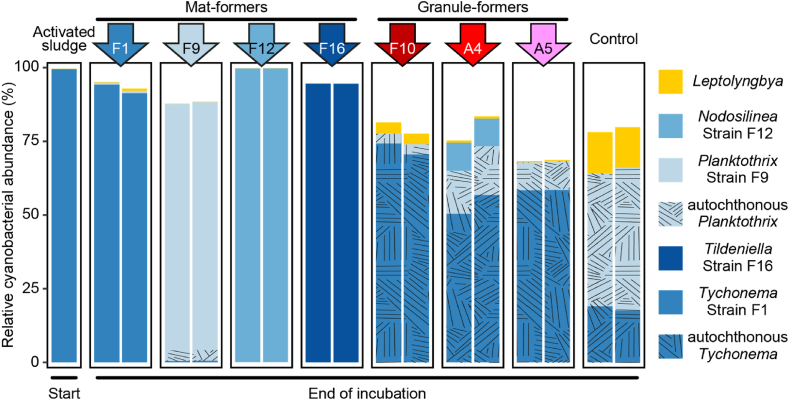
Relative abundance of major cyanobacterial OTUs among phototrophic sequences in the initial activated sludge and at the end of Experiment 10. The control represents unaugmented activated sludge after incubation. Augmented strains are indicated by colored arrows above the bar plots, with each bar representing the cyanobacterial community in a single vial (either photogranules or mats). Strains added and those detected in the final morphotypes are color-coded as shown in the legend. Blue tones represent OTUs of mat-forming strains (F1, F9, F12, F16). Hatched patterns denote autochthonous OTUs from the same genera as the augmented strains, likely originating from the activated sludge. Note the absence of the granule-forming strains in the final communities. (For interpretation of the references to color in this figure legend, the reader is referred to the Web version of this article.)

**Fig. 4:**
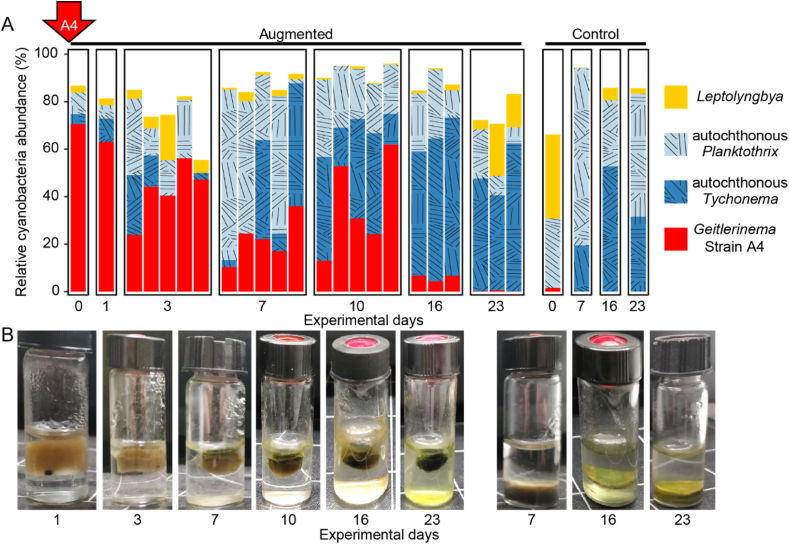
Cyanobacteria succession over time and representative pictures of morphology progression of augmented biomass (A) and control biomass (B) (experiment 2). Each bar represents one sample. No quantitative analysis was done, hence comparisons in composition between control and augmented samples and between different days need to be carefully considered, since total amount of cyanobacteria must change.

Our imaging approach is limited by floating biomass, i.e., a floating sludge bed early in the experiment, or floating photogranules at later stages of the experiment [3,12,21]. This phenomenon may occur when for example supersaturated oxygen degasses from the water phase and attaches as fine bubbles at the granule surface or forms larger bubbles inside the biomass. Because of the increased buoyancy, the biomass floats and moves too far out of the scanner's focal plane. On the resulting images, the unfocussed biomass appears to cover a larger area than it actually does, creating a visual artifact that gives the impression of lack of contraction. We did not remove the data derived from these images from Fig. 2, but put the interpretation of the image data into perspective. As highlighted by Zimmer [22] for automated experiments, the quality control of image data and data interpretation by a human expert is required to avoid misinterpretation. In the current case, quality checking allowed us to identify floating of the initial sludge bed in experiment 11 for strains A5 and F10. Biomass of strain F10 in experiment 11 additionally resettled and floated again when photogranules were formed (Fig. 2, Experiment 11). In experiment 10, the initial sludge augmented with F9 was partially attached to the bottom of the vials while part of the biomass floated. This behavior created an effect in the scanner data similar to contraction (Fig. 2). Later, all biomass floated covering the entire vial diameter as observed (F9 in Fig. 1).

Between the various incubation sets, differences can be detected, even when the same strains were used. Note for example the augmentations with strain F10. All four incubation sets using F10 (experiments 4, 5, 10 and 11) finish at a comparable end point, but the kinetics of photogranulation differ. Despite these differences, per incubation set, the small confidence intervals (shaded areas in Fig. 2) signify a low variability within incubations of the same set. Wider confidence intervals can be observed for experiments 10 and 11 when fewer vials per augmentation series were incubated. Nonetheless, all replicates in the same sets develop the same morphotype, either mats or photogranules. These observations justify the assumption that biomass in all vials behaves comparably. Therefore, any vial can be sacrificed for sampling at any position in the experimental layout and any sampling time. The cyanobacteria present in samples taken at intermediate time points during an incubation therefore represent transient stages in a succession of community members forming a final morphotype.

### 3.3 Microbial composition before and after augmentation

Two experiments (10 and 11) were conducted in which the behavior of all seven strains was compared in the same experiment. Experiments 10 and 11 used two different sources of activated sludge. Also control vials containing only activated sludge developed phototrophic communities, however with an unconsolidated, sludge like consistency (Fig. 1). With the parallel incubations using all cyanobacterial strains at once, we can rule out any potential variability introduced in sequential incubations from different batches of activated sludge.

For experiment 10, the activated sludge community before augmentation was sequenced, as well as the microbial communities once the incubations reached their final morphotype. The relative abundances of cyanobacterial sequences among all phototrophic sequences present in the 23S rRNA amplicons are shown in Fig. 3. The cyanobacterial proportion among the total bacteria of the initial activated sludge represents a very small population. The interpretation of the initial activated sludge needs to be done with caution, as the random presence of a cyanobacterial type may drastically change the visual impression of the sample in the figure. Thus, the initial cyanobacterial community is susceptible to drastic changes based on the amount of cyanobacteria added to the activated sludge.

Two general scenarios were identified at the end of the incubations: when mat-forming strains were added (F1, F9, F12 and F16), the added strains became predominant in the final communities (Fig. 3, “Mat-formers”). The addition of a mat-former had a marked influence on the final community and the cyanobacteria initially present in the activated sludge were outcompeted. An exception is the F1 addition. An OTU with the F1 sequence was already detected in the initial activated sludge. In the second scenario, when granule-forming strains were added (A4, A5 and F10), we were unable to detect these strains in the final communities at the end of the incubation. Amplification bias and different rRNA operon copy numbers may influence these results at the lower end of the detection limit. A more targeted qPCR approach with strain-specific primers would provide even stronger results but was not available at the time of the study. Instead, the phototrophic populations are dominated by two cyanobacterial OTUs of the genera *Tychonema* and *Planktothrix with* 99.7 % and 95.4 % nucleotide identity with the mat-forming strains F1 and F9, respectively. These organisms may also represent so far uncharacterized mat-formers. Generally, the cyanobacterial populations were more diverse after successful photogranule formation, as can be seen in the larger proportion of minor cyanobacterial OTUs in the samples (white complements of bars to reach 100 %).

The strong decrease of the granule-forming strains after photogranule formation was also observed by Joosten et al. [13] and is confirmed here for two additional strains that were not tested before. Thus mat-formers became the most abundant cyanobacteria, independent of the actual final morphotype. Nevertheless, even though the added strains were undetected in the final communities, their impact was obvious in the developed morphotypes and in the difference between control communities and augmented communities.

### 3.4 Population dynamics of cyanobacteria during photogranulation

The experimental approach that we introduce here allows us to follow the microbial succession of the cyanobacteria population during photogranulation and not only at the end point, because we were able to demonstrate the reproducibility of morphotype development in replicates. To deepen our understanding of the microbial succession during photogranulation, we analyzed the intermediate cyanobacterial populations after augmentation with the granule-forming strain A4. As expected, the incubation yielded photogranules in all vials that remained after the sacrificial sampling for sequencing (Experiment 2 in Fig. 2).

The cyanobacterial populations determined from the 23S rRNA amplicons at sampling days 0, 1, 3, 7, 10, 16 and 23 are presented in Fig. 4. At day 0, the augmented strain A4 initially represented 70.5 % of all cyanobacterial sequences in the augmented sludge. Though decreasing in relative abundance over time, it remained detectable in all tested photogranules until day 16 after which it eventually fell below the detection limit. Ideally, an absolute quantification, e.g., by qPCR, would be desirable to measure true decreases in absolute numbers and not just relative decreases that could also be caused by increases of other cyanobacteria.

**Fig. 5:**
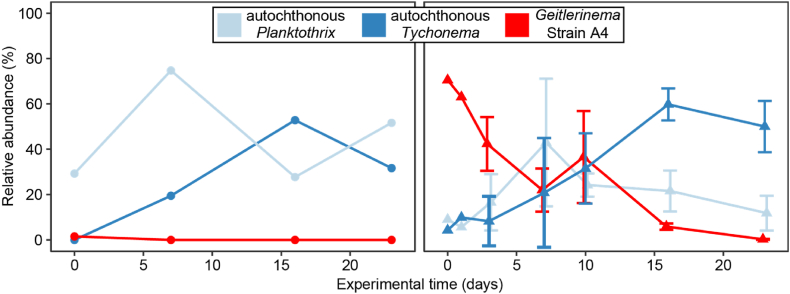
Focus on the strain *Geitlerinema* sp. A4 (red), autochthonous *Planktothrix* (light blue) and autochthonous *Tychonema* (dark blue) in developing phototrophic ecosystems. On the left as filled circles the control experiment without the addition of A4, maintaining an unconsolidated, sludge-like matrix and on the right as triangles after augmentation with A4, developing photogranules. (For interpretation of the references to color in this figure legend, the reader is referred to the Web version of this article.)

A *Planktothrix* OTU transiently increased over time with a peak in relative abundance on day 7. Its abundance consequently decreased significantly and reached again its initial abundance at the end of the incubation. A *Tychonema* sequence type was systematically found in all photogranules sampled after day 7, increasing in relative abundance and dominating the population until the end of the experiment. This succession of the three cyanobacteria is emphasized in Fig. 5. We contrast the behavior in the augmented vials with the unaugmented control vials, where no A4 was added. As the total phototrophic population likely increased in absolute numbers over time, a decrease in A4 may not necessarily mean a disappearance of A4 but rather its dilution by a disproportionately faster growth of *Tychonema* and *Planktothrix* over time.

The photogranule morphotype was first detectable on day 7 (Fig. 4B) when strain *Geitlerinema* sp. A4 was still the most abundant cyanobacterium in the population. Even when A4 disappeared from the sequence inventories, the established photogranule morphotype remained in place. This happened despite the increase in relative abundance of *Planktothrix* sp. and *Tychonema*, both previously classified as mat-forming cyanobacteria. The elevated relative abundance of mat-forming cyanobacteria in the second half of the incubation did not induce the development of a mat morphotype. We hypothesize instead that A4, when it was still present, engineered the ecosystem towards photogranule formation and maintained its impact even after its strong reduction in relative abundance. In the literature, cyanobacteria of the genus *Geitlerinema* were already detected in photogranules at the end of hydrostatic incubations at low abundance compared to other filamentous cyanobacteria [3]. Because of this low abundance, they attracted no particular attention. However, considering a succession of cyanobacteria during photogranulation, a low abundance in the final phenotype does not allow any conclusion about the importance of this bacterial group at an earlier developmental state of the ecosystem. Likewise, the high relative abundance of certain cyanobacteria at the end of the incubation does not necessarily proof their role in the morphotype formation as was implied in earlier work [3,8]. A comparable trend may have been observed in cryoconite particles. Cryoconites are photogranule-like structures found on glacier surfaces that share features with photogranules produced in the context of wastewater treatment [9]. A diversity of phototrophic microorganisms can be observed in cryoconite particles, but the larger cryoconite particles grow, the more cyanobacteria belonging to the *Oscillatoria* dominate the community [23]. This may, as in our case here, also point towards a competitive genus of cyanobacteria that overtakes the community with time. Future experiments would strongly benefit from an absolute quantification of abundance of mat- and photogranule-formers.

**Fig. 6:**
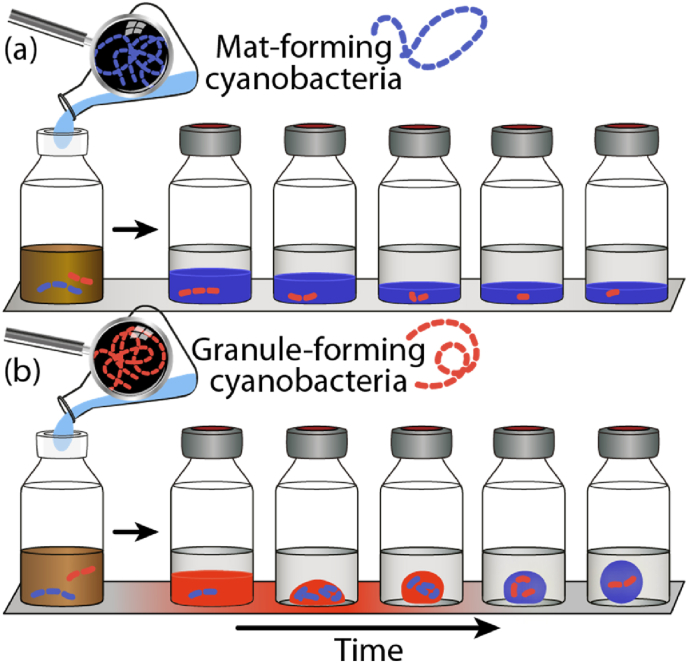
Schematic representation of the effect of adding mat- and granule-forming cyanobacteria to activated sludge. (a) The addition of sufficient quantities of mat-formers (blue) leads to the development of a mat morphotype, while granule-forming cyanobacteria are undetectable or present in small numbers. (b) The addition of sufficient quantities of granule-formers (red) leads to a photogranule morphotype that is, however, also dominated by mat-forming cyanobacteria while the added granule-formers are undetectable or present in small numbers. The red-shaded floor indicates the putative window of opportunity in which activity and presence of granule-forming cyanobacteria is highest. (For interpretation of the references to color in this figure legend, the reader is referred to the Web version of this article.)

A4 is undetectable in the final morphotype here, but it may have left its trace in the spatial organization of the microbial community. The changes putatively induced by A4 persisted under the tested conditions. Thus, the augmentation with A4 overcame the resilience of the ecosystem to return to its original trajectory of morphotype development which would have resulted in a mat-like morphotype. The phenomenon is schematically illustrated in Fig. 6. The phenomenon of changing an ecosystem trajectory, even though the causal agent disappears over time, has also been observed for other ecosystems, e.g., soil [24]. At the same time, we need to wonder how the created structure is preserved or even maintained when the putative organism that causes it, here the strain A4, is becoming rare in sequence inventories of the population and cannot any longer exert its immediate impact on photogranulation. A parsimonious explanation may be that the trichomes of the initial granule formers constitute a scaffold that aligns newly grown motile filamentous cyanobacteria and lets them copy and thus maintain the already established structure. Even mat-forming, motile filamentous cyanobacteria that are unable to create the initial structure themselves may nonetheless be able to maintain it. Auto-alignment of trichomes is a known property of motile filamentous cyanobacteria [6]. Non-gliding filamentous cyanobacteria should not be able to auto-align and may be able to passively disrupt the photogranule morphotype if they gain dominance of the cyanobacterial population, providing an intriguing hypothesis for future experiments.

Changing the trajectory of ecosystem development may have implications for the inoculation of photobioreactors for biotechnological applications. Mature hydrostatic photogranules similar to the final morphotypes produced here are often used as inoculum [25,26]. However, in the mature state, the responsible organisms for photogranulation may already be present at low abundance and possibly low activity. A higher abundance and higher activity of the photogranule-forming cyanobacteria may be found in hydrostatic photogranules in a short window of opportunity at intermediate states of morphotype development. In our case, this may have been between days three to ten in our 25-days incubation period. It remains to be seen if photogranulation in hydrodynamic environments also follows a microbial succession.

Even though a cyanobacterial succession was demonstrated in this study in which the strain *Geitlerinema* sp. A4 eventually fell below the detection limit, the time when it was present was sufficient to induce photogranulation. Other environmental conditions, like the temperature of incubation, may change the competitive behavior of strain A4 and consequently the final morphotype. This temperature dependence was observed in Castro et al. [4], where two sludge sources placed in hydrostatic incubations switched in morphotype from mat formation at lower temperature to photogranule formation at higher temperature of incubation. Only considering the abundance of a photogranule-former may thus be a simplification of a more complicate interplay between community composition and environmental conditions. Photogranulation would thus depend on both the presence of an organism and the conditions that enable it to dominate and potentially outcompete autochthonous populations. The reported temperature-dependence of the switch in morphotype was associated with a change in abundance of cyanobacteria and in the concentration and composition of EPS, with more polysaccharide-enriched EPS in mats compared to photogranules. The effects of cyanobacteria density, or the role of EPS, are still under debate since other studies showed opposite results [3]. The data in the literature therefore indicate contradictory results that warrant further investigation.

When attempting to identify the causal agents of photogranulation, it may be wise to extend the search to the period that we identified in Fig. 6 for sampling. The postulated highest activity of the ecosystem engineer may occur in this short window of time during the first half of the incubation period, possibly even before the final morphotype becomes obvious.

## 4 Conclusions

When adding a comparably small amount of cyanobacteria to activated sludge, we were able to reproducibly override the inherent properties of the autochthonous cyanobacterial population: we were able to force an activated sludge matrix to form a mat- or photogranule morphotype.

We demonstrated the transient presence of the cyanobacteria that we believe to be responsible for photogranule formation. However, these photogranule formers were absent in the final cyanobacterial populations that were dominated, irrespective of the morphotype, by what we classify as mat-forming cyanobacteria.

This transient presence of the ecosystem engineer has immediate consequences for how we imagine the life cycle of photogranules. Decisive steps towards photogranulation may already be taken before the morphotype is visually detectable. Studying the mechanism of photogranulation thus requires a focus on the beginning of the incubation and less on the final product.

Hydrostatic photogranules with the intended use as inoculum for starting up photobioreactors harboring hydrodynamic photogranules may best be harvested at intermediate states of morphotype development to maximize presence and activity of photogranule forming cyanobacteria.

## CRediT authorship contribution statement

**Sandra Galea-Outón:** Writing – original draft, Visualization, Methodology, Formal analysis, Conceptualization. **Jérôme Hamelin:** Writing – review & editing, Validation, Supervision, Methodology, Conceptualization. **Kim Milferstedt:** Writing – review & editing, Visualization, Validation, Supervision, Project administration, Methodology, Conceptualization.

## Funding

Région Occitanie and INRAE division MICA.

INRAE Metaprogram Holoflux

## Declaration of competing interest

The authors declare the following financial interests/personal relationships which may be considered as potential competing interests:Sandra Galea-Outon reports financial support was provided by Région Occitanie. If there are other authors, they declare that they have no known competing financial interests or personal relationships that could have appeared to influence the work reported in this paper.

## Data Availability

Data will be made available on request.
