# Supplementary material for: Transient cyanobacteria drive hydrostatic photogranulation but disappear during microbial succession
Source: Biofilm. 2025 Dec 1;11:100339. doi: 10.1016/j.bioflm.2025.100339 (PMC12721295; doi:10.1016/j.bioflm.2025.100339)
Supplement: Multimedia component 1 [file mmc1.pdf]

Supplementary material

Transient cyanobacteria drive hydrostatic photogranulation but disappear during microbial succession

Sandra Galea-Outón[1], Jérôme Hamelin, Kim Milferstedt

INRAE, Univ Montpellier, LBE, 102 Avenue des étangs, 11100, Narbonne, France.

Corresponding author: Kim Milferstedt

e-mail: kim.milferstedt@inrae.fr

Current address: sandra.galea@uvic.cat, BETA Technological Center Futurlab, Can Baumann

Ctra de Roda 70, 08500 Vic, Spain

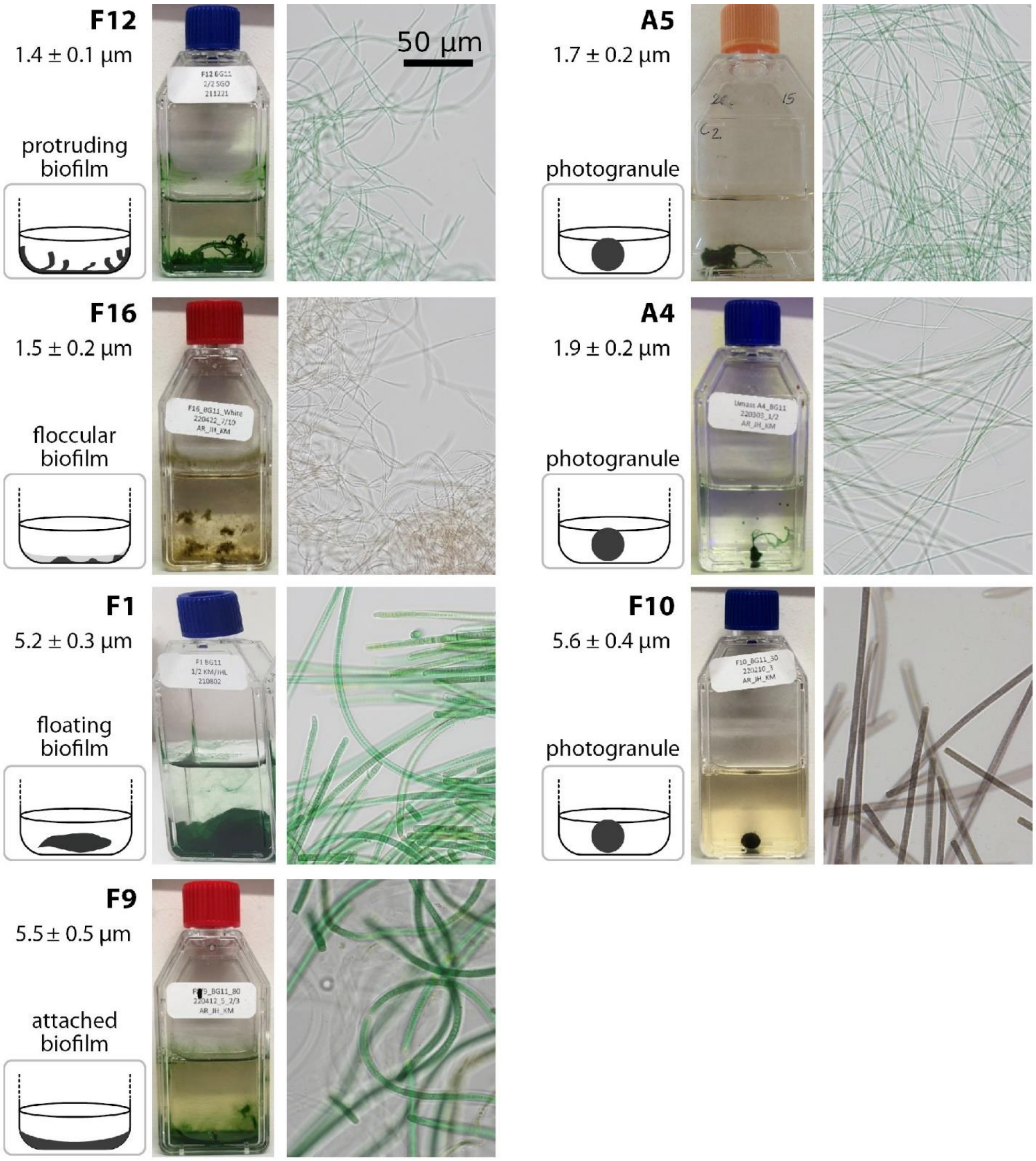

Figure S 1: Morphological characterization of monoclonal cultures of the seven cyanobacterial strains use in this study (A4, A5, F10, F1, F9, F12 and F16), including the average filament diameter, a macroscopic picture of the culture in the culturing flask, a schematic representation of its morphotype, and a brightfield microscopic image at the same scale for all strains.

Table S 1: Names of samples that were used to produce **Error! Reference source not found.** and **Error! Reference source not found.**. The samples can be found at the European Nucleotide Archive under the accession number PRJEB91337.

Figure	Identifier	Sample name in Bioproject
3	AS (activated sludge)	Orn_23S
	A4	End_A4_Orn_1_23S
		End_A4_Orn_2_23S
	A5	End_A5_Orn_1_23S
		End_A5_Orn_2_23S
	F10	End_F10_Orn_1_23S
		End_F10_Orn_2_23S
	F12	End_F12_Orn_1_23S
		End_F12_Orn_2_23S
	F16	End_F16_Orn_1_23S
		End_F16_Orn_2_23S
	F1	End_F1_Orn_1_23S
		End_F1_Orn_2_23S
	F9	End_F9_Orn_1_23S
		End_F9_Orn_2_23S
	Control	End_Orn_1_23S
		End_Orn_2_23S
4A	Augmented day 0	231120_Aug_23S
	Augmented day 1	231121_Aug_2_23S
	Augmented day 3	231123_Aug_1_23S
		231123_Aug_2_23S
		231123_Aug_4_23S
		231123_Aug_5_23S
		231123_Aug_6_23S
	Augmented day 7	231127_Aug_1_23S
		231127_Aug_2_23S
		231127_Aug_3_23S
		231127_Aug_4_23S
		231127_Aug_5_23S
	Augmented day 10	231130_A4_Orn_1_23S
		231130_A4_Orn_2_23S
		231130_A4_Orn_3_23S
		231130_A4_Orn_4_23S
		231130_A4_Orn_5_23S
	Augmented day 16	231206_A4_Orn_1_23S
		231206_A4_Orn_2_23S
		231206_A4_Orn_3_23S
	Augmented day 23	231213_Aug_1_23S
		231213_Aug_2_23S
		231213_Aug_3_23S
	Control day 0	231120_Sludge_23S
	Control day 7	231127_Sludge_23S
	Control day 16	231206_Control_Orn_nd_23S
	Control day 23	231213_Sludge_23S
